# Functional mandibular reconstruction using digital planning-guided titanium scaffolds combined with vascularized fibula flaps

**DOI:** 10.1093/rb/rbag181

**Published:** 2026-08-18

**Authors:** Huawei Liu, Shuaiyin Liu, Yongfeng Li, Weisheng Wen, Qi Ning, Lingling Zheng, Chao Wang, Juncheng Wang, Min Hu

**Affiliations:** Medical School of Chinese PLA, Beijing 100853, China; Department of Stomatology, The First Medical Center, Chinese PLA General Hospital, Beijing 100853, China; School of Engineering Medicine, Beihang University, Beijing 100083, China; Key Laboratory of Biomechanics and Mechanobiology, Beihang University, Ministry of Education, Beijing 100191, China; Key Laboratory of Innovation and Transformation of Advanced Medical Devices, Ministry of Industry and Information Technology, Beihang University, Beijing 100191, China; National Medical Innovation Platform for Industry-Education Integration in Advanced Medical Devices, Interdiscipline of Medicine and Engineering, Beihang University, Beijing 100191, China; School of Biological Science and Medical Engineering, Beihang University, Beijing 100191, China; Department of Stomatology, Beijing Tsinghua Changgung Hospital, School of Clinical Medicine, Tsinghua University, Beijing 102218, China; Medical School of Chinese PLA, Beijing 100853, China; Department of Stomatology, The First Medical Center, Chinese PLA General Hospital, Beijing 100853, China; Medical School of Chinese PLA, Beijing 100853, China; Department of Stomatology, The First Medical Center, Chinese PLA General Hospital, Beijing 100853, China; Key Laboratory of Biomechanics and Mechanobiology, Beihang University, Ministry of Education, Beijing 100191, China; Key Laboratory of Innovation and Transformation of Advanced Medical Devices, Ministry of Industry and Information Technology, Beihang University, Beijing 100191, China; National Medical Innovation Platform for Industry-Education Integration in Advanced Medical Devices, Interdiscipline of Medicine and Engineering, Beihang University, Beijing 100191, China; School of Biological Science and Medical Engineering, Beihang University, Beijing 100191, China; Key Laboratory of Biomechanics and Mechanobiology, Beihang University, Ministry of Education, Beijing 100191, China; Key Laboratory of Innovation and Transformation of Advanced Medical Devices, Ministry of Industry and Information Technology, Beihang University, Beijing 100191, China; National Medical Innovation Platform for Industry-Education Integration in Advanced Medical Devices, Interdiscipline of Medicine and Engineering, Beihang University, Beijing 100191, China; School of Biological Science and Medical Engineering, Beihang University, Beijing 100191, China; Medical School of Chinese PLA, Beijing 100853, China; Department of Stomatology, The First Medical Center, Chinese PLA General Hospital, Beijing 100853, China; Medical School of Chinese PLA, Beijing 100853, China; Department of Stomatology, The First Medical Center, Chinese PLA General Hospital, Beijing 100853, China

**Keywords:** scaffold-driven DBFF, titanium scaffolds, mandibular bone defects, stress distribution

## Abstract

Segmental mandibular reconstruction presents a significant clinical challenge that requires addressing structural defects while restoring proper masticatory function and facial esthetics. To optimize this procedure, a novel digital–biomechanical, scaffold-driven double-barrel fibula flap (DBFF) strategy was established and validated using patient-specific, 3D-printed porous titanium scaffolds. The study evaluated three distinct defect models (hemimandibular, central and lateral) through finite element simulations at various postoperative stages under biting forces, alongside a retrospective clinical analysis of 16 patients with a mean follow-up of 15.2 months. The results demonstrated successful progressive load transfer from the scaffold to the fibula, preserved temporomandibular joint biomechanics and marked intra-scaffold osteogenesis. Ultimately, this approach achieved accurate mandibular reconstruction, early dental rehabilitation and high patient satisfaction without mechanical failure or scaffold exposure, concluding that this digitally guided, scaffold-assisted DBFF strategy offers a safe, functional and highly promising solution for clinical application.

## Graphical abstract

**Graphical Abstract:**
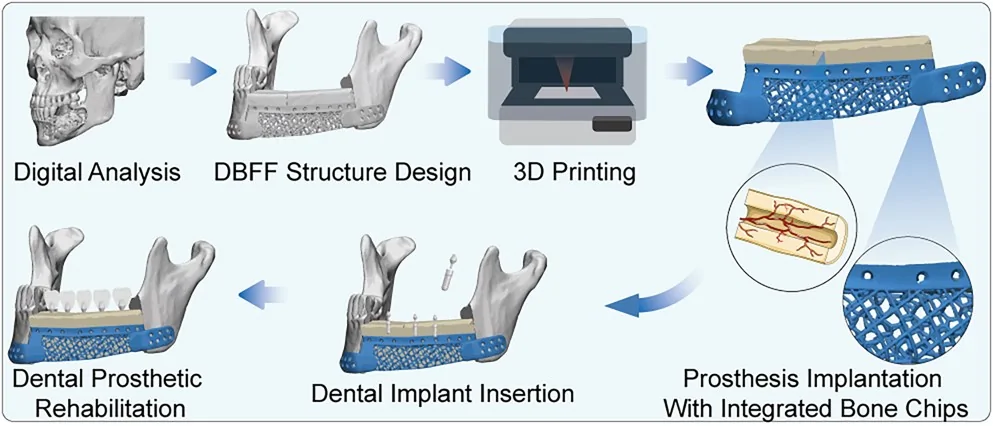

## Introduction

Segmental mandibular defects typically arise from trauma, benign or malignant mandibular tumors or segmental mandibular resection for eradicating mandibular osteomyelitis [1, 2]. Failure to achieve functional repair may lead to impaired mastication, deglutition and phonation, alongside psychological sequelae secondary to facial deformities [3, 4]. Consequently, autogenous bone grafting remains the gold standard for mandibular reconstruction [5], with vascularized fibula flaps emerging as the predominant surgical approach. This technique provides bicortical bone coverage and robust vascular supply, particularly critical for large-scale defects (>10 cm) [6].

Recent advancements in medical technology have further shifted the paradigm from mere functional restoration to comprehensive rehabilitation integrating esthetics and occlusal function [7, 8]. To meet these evolving clinical demands, individualized digital design has become a mainstream method for repairing mandibular bone defects, which requires sufficient bone grafts for dental implants [9, 10].

However, the vertical height discrepancy between the native mandible and fibular graft poses significant challenges for dental implantation [11]. While single-strut fibular reconstruction has been conventionally employed for mandibular defect repair, its clinical limitations include suboptimal facial contour restoration, stress concentration at the implant interface and elevated failure rates [12]. To address these shortcomings, the double-barrel fibula flap (DBFF) technique has been developed to provide augmented bone volume, thereby enabling functional rehabilitation through denture restoration with improved facial esthetics, occlusal function and speech/swallowing recovery [13, 14]. Despite its acceptable esthetic and functional outcomes, several inherent drawbacks of DBFF have been reported. First, it is difficult to bridge mandibular defects larger than 9 cm due to the limited length of the fibula. Second, surgical procedures of double-barreling, vascular pedicle folding and vascularized fibular graft implantation are time-consuming, leading to an increased risk of postoperative complications. Third, the double-barrel graft may result in excessive bone height that shortens the interridge distance, thus hindering subsequent dental implantation.

Given these limitations of conventional DBFF, 3D-printed porous titanium implants have emerged as a promising alternative, demonstrating significant potential for reconstructing large-segment bone defects and augmenting osteogenesis. Compared with polymer-based scaffolds, titanium alloys offer significantly superior mechanical strength, fatigue resistance and long-term structural stability, which are essential for load-bearing reconstruction of large segmental mandibular defects. In addition, titanium exhibits excellent biocompatibility, corrosion resistance and favorable osseointegration properties, enabling stable support for bone regeneration under functional loading conditions. These advantages make titanium-based porous scaffolds particularly suitable for craniofacial reconstruction where immediate mechanical integrity and long-term durability are required. Schouman *et al.* [15] validated that stiffness-optimized porous titanium alloy scaffolds exhibit superior osteoconductive properties, particularly in facilitating bone tissue ingrowth. Similarly, Pobloth *et al.* [16] validated that when implanted in the long bones of 27 sheep, a porous mesh scaffold combined with autologous bone particles can effectively promote early bone defect bridging, endochondral bone formation and bone regeneration in critical-size bone defects. However, previous explorations have mainly focused on the local osseointegration at the interface between the implant and the jawbone, with only a low level of bone regeneration appearing within the implant. This underscores a critical challenge: achieving complete intra-scaffold osteogenic repair remains elusive. Notably, our prior study [17] provides preliminary evidence of localized bone regeneration within canine mandibular segmental defects following 6-month implantation of 3D-printed mesh titanium prostheses, suggesting a promising pathway to address this limitation.

Building upon these findings, the integration of 3D-printed titanium alloy implants with fibula grafts offers the potential to reshape mandibular morphology while overcoming the limitations of conventional DBFF. Nevertheless, most previous studies are case reports lacking systematic analysis of their mechanical properties and potential risks [18]. To address this critical gap, our team pioneered a scaffold-driven DBFF technique combining 3D-printed porous titanium scaffolds with vascularized fibular flaps for functional mandibular reconstruction [19]. This innovative approach offers multiple advantages, including reduced fibular usage, shorter operative duration for osteotomy and vascular anastomosis, immediate dental implantation capability and fewer mechanical complications. However, comprehensive biomechanical evaluation and robust clinical validation of this technique remain limited, necessitating systematic investigation of its long-term performance and safety.

Therefore, in the present study, we established three representative mandibular defect models based on the HCL classification: (1) hemimandibular segment (condyle-inclusive, HS), (2) central segment (canine-inclusive, CS) and (3) lateral segment (condyle-exclusive, LS). Each model was reconstructed using 3D-printed porous titanium scaffolds (strut diameter: 0.5 mm; pore diameter: 5.0 mm) combined with vascularized fibular flaps. Finite element analysis was performed to systematically evaluate biomechanical performance at three critical time points (immediate post-implantation, 6 months pre-dental rehabilitation and 9 months post-dental rehabilitation) under both physiological chewing force (200 N) and maximum bite force (800 N). Furthermore, clinical outcomes were prospectively assessed in 16 patients with mean follow-up of 15.2 months, evaluating reconstruction accuracy, osseointegration, functional recovery and patient satisfaction. These findings aim to provide comprehensive evidence-based support for broadening the translational application of scaffold-driven DBFF in mandibular reconstruction (Figure 1).

**Figure 1:**
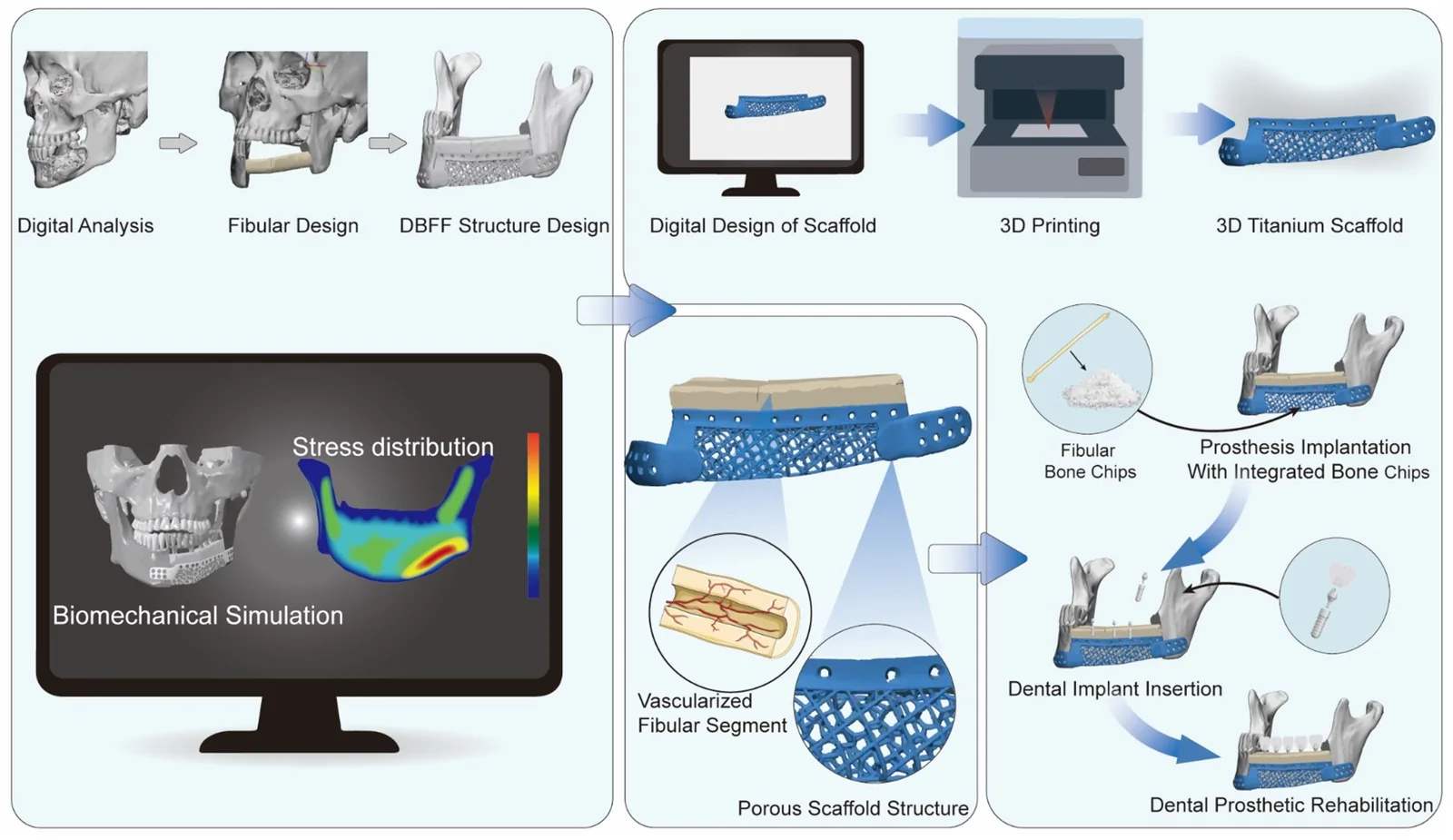
Digital workflow and design framework of scaffold-driven DBFF reconstruction. The workflow includes preoperative CT-based 3D reconstruction of mandibular defects, digital planning of fibular osteotomy guided by three key geometric parameters (L1, L2 and α) derived from the contralateral mandible and the design of a two-layer reconstruction system consisting of a superior vascularized fibula for functional load restoration and an inferior 3D-printed porous titanium scaffold for contour reconstruction. Autologous bone grafts are incorporated into the large-pore scaffold to facilitate intra-scaffold osteogenesis and a final virtual surgical simulation defines the mandibular morphology, fixation strategy and implant positioning.

## Materials and methods

### Three-dimensional reconstruction and preoperative digital design

Spiral CT data of the mandible and fibular specimens were imported into Mimics 14.12 (Materialise, Leuven, Belgium) for 3D reconstruction and surface smoothing. Based on tumor characteristics and clinical resection principles, an osteotomy line was designed with appropriate safety margins around the mandibular lesion. Following the HCL classification system, three mandibular defect models were constructed: hemimandibular segment involving the condyle (HS), central segment encompassing the canine teeth (CS) and lateral segment excluding the condyle (LS). The reconstruction system employed a dual-layer architecture: (1) a superior vascularized fibula flap for bone defect regeneration and subsequent implant-supported dental rehabilitation and (2) an inferior 3D-printed porous titanium scaffold (strut diameter: 0.5 mm; pore diameter: 5.0 mm; porosity: ∼90%) for initial mechanical load-bearing and anatomical contour restoration. The scaffold design incorporated a biomimetic structure with an external cortical-like shell and internal trabecular-like porous network to facilitate autologous bone graft integration. Fibular osteotomy planning was guided by three key geometric parameters (L1, L2 and angle α) derived from contralateral healthy mandibular anatomy to ensure optimal morphological restoration. The mandibular fixation plate extended from the buccal surface of the prosthesis to the native mandible, while the fibular plate connected the prosthesis to the lateral fibular surface. Following scaffold placement, excess fibular bone tissue was milled into particulate form and used to fill the scaffold’s porous network to promote volumetric osteogenesis. Virtual surgical simulation was performed to finalize mandibular morphology, fixation strategy and dental implant positioning (Figure 1).

### Finite element analysis

Finite element analysis was conducted to evaluate stress, strain and deformation in the mandible, fibula, titanium scaffold and condylar articular disk. The models were generated and analyzed using ANSYS software (Swanson Analysis, TX, USA) at three time points: immediately after scaffold implantation, 6 months post-implantation (before denture rehabilitation) and 9 months post-implantation (after denture rehabilitation). All materials were assumed to be homogeneous, isotropic and linearly elastic. The finite element models before and after denture restoration, as well as the Young’s modulus and Poisson’s ratio of each material (cortical bone, cancellous bone, articular disk, tooth, dental implant, screw, fibula, titanium scaffold and bone graft), are listed in Table 1 [19].

**Table 1:** Mechanical properties of the materials used in the functional repair of mandibular bone defects.

Materials	Young’s modulus (MPa)	Poisson’s ratio
Cortical bone	15 000	0.3
Cancellous bone	1500	0.3
Articular disk	44.1	0.4
Tooth	20 000	0.3
Dental implant	110 000	0.34
Screw	110 000	0.34
Fibula	15 000	0.3
Titanium scaffold	110 000	0.34
Bone grafting material filling (immediately after implantation)	1000	0.3
Bone grafting material filling (6 months of implantation)	5000	0.3

In addition, the mandibular movement was simulated by applying the muscle force with varying directions at the maximum bite force of 800 N and chewing bite force of 200 N. The muscle force values and the directions of the muscle structures were obtained as previously reported. They were evenly distributed on nearby nodes where the load was located, based on the muscle region. The muscle force distributed in the eight regions on bilateral sides is shown in the existing literature [15, 20].

### A digital navigation system used in one-stage functional repair of mandibular bone defects and dental implantation

Under general anesthesia via nasotracheal intubation, the defective mandibular bone was removed with the assistance of an osteotomy in an extraoral approach. The 3D-printed titanium scaffold was placed and fixed to restore the occlusion. Osteotomy and shaping of the fibula were then conducted. Fibular flaps dissected off the vascular pedicle were fixed on the mandible, where the titanium scaffold served as a plate to guide the position. A 1-mm space superior to the titanium scaffold on the lingual side was designed to allow fine adjustments of the fibula. Microscopic anastomosis of the peroneal and facial arteries and venous anastomoses of the peroneal, retromandibular and external jugular veins using a vascular stapler were completed. The blood flow of the implanted fibula was examined. Immediately after the repair of mandibular bone defects, dental implantation was conducted under the guidance of the digital navigation system (No. Remebot RM-200) developed by our team. Finally, the excess fibula was ground to fill the gaps in the scaffold. The workflow of digital-assisted one-stage functional repair of mandibular bone defects and dental implantation is shown in Figure 2.

**Figure 2:**
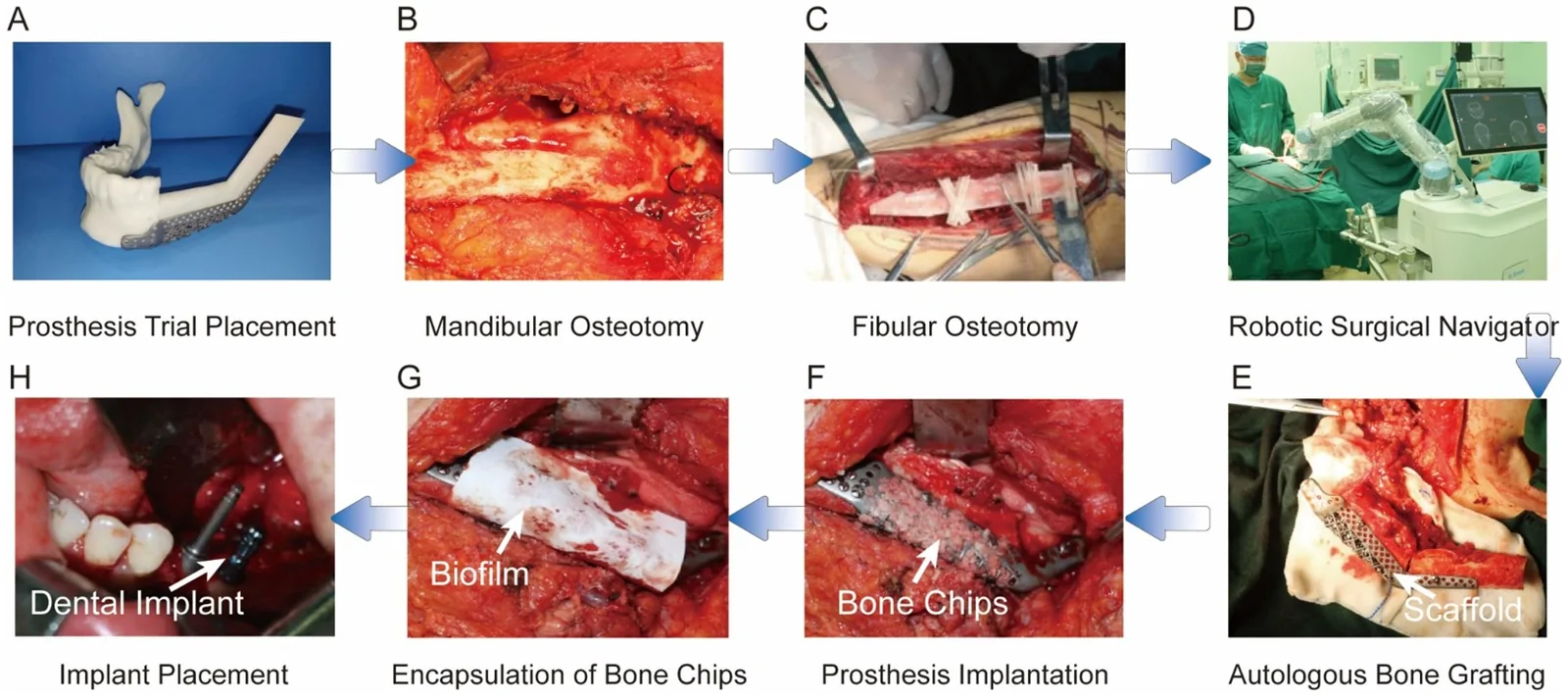
The workflow of digital-assisted one-stage functional repair of mandibular bone defect and dental implantation. (**A**) Preoperative scaffold fitting and verification of mandibular alignment. (**B**) Tumor exposure and preservation of the inferior alveolar neurovascular bundle. (**C**) Guided fibular harvest and shaping while preserving the vascular pedicle. (**D**) Real-time dental implant placement using robotic digital navigation. (**E**) Fibular vascular anastomosis and placement of the 3D-printed titanium scaffold as an intraoperative positioning guide. Dental implantation guided by intraoperative digital navigation system. (**F**) Autologous fibular bone chips filling the scaffold pores to promote volumetric osteogenesis. (**G**) Bone fragments encapsulated by biofilm. (**H**) Implant implantation.

### Perioperative infection prevention and postoperative monitoring

All patients received standardized perioperative infection prophylaxis. Intravenous broad-spectrum antibiotics were administered prior to incision and repeated intraoperatively when surgical duration exceeded 3 h to maintain effective antimicrobial coverage. Strict aseptic surgical techniques and meticulous debridement were applied in all cases. During reconstruction, autologous fibular bone was processed into particulate grafts and tightly packed into the porous structure of the titanium scaffold to eliminate potential dead space and enhance local tissue integration. This may contribute to a stable biological environment and reduce the risk of postoperative infection. Postoperatively, patients underwent routine wound care combined with effective negative-pressure drainage. Routine blood tests and C-reactive protein levels were monitored for three consecutive days, and microbiological culture of drainage fluid was performed when clinically indicated to enable early detection of potential infection.

### Postoperative assessment of the accuracy of the implant placement

CT images were captured at 1 week and 6 months postoperatively for 3D reconstruction. By comparing the postoperative CT scans with the preoperative digital design, the accuracy of mandibular bone defect repair by 3D-printed titanium scaffolds combined with autologous fibula flaps was assessed. Conditions at a total of 10 positions, involving five anatomical landmarks (condyle, midpoint of the posterior margin of mandible, angle of the mandible, midpoint of the inferior margin of mandible, coracoid) on bilateral sides were compared.

### Postoperative follow-up

The final repair with dental implantation was conducted at 6–9 months postoperatively. Postoperative satisfaction, osteogenic performance (osteogenesis at the junction of fibula and mandible, internal osteogenesis in the repaired mandible) and long-term complications (screw loosening, infection, exposure of the scaffold/implantation) were assessed. To determine objective evaluations, facial symmetry, facial height, articulation, chewing, swallowing and mandibular movement were evaluated using a 5-point Likert scale, where 1 point was very abnormal and 5 points were completely normal [21].

### Statistical analysis

Statistical analysis was performed using SPSS version 25 (IBM, Chicago, IL, USA). The Kolmogorov–Smirnov and Shapiro–Wilk tests were used to assess the normality distribution. If the variable followed a normal distribution, a paired *t*-test was conducted. If not, the Wilcoxon signed-rank test was performed. Statistical significance was defined as a *P* values < 0.05. Multiple levels of significance were indicated as ns (not significant), *P < 0.05, **P < 0.01 and ***P < 0.001.

## Ethical approval

The study was approved by the Ethics Committee of Chinese PLA General Hospital (Approval No. S2023-396-01).

## Informed consent

Written informed consent was obtained from all individual par-ticipants included in the study.

## Results

### Design and biomechanical optimization of scaffold-driven DBFF

To establish a rational design framework for scaffold-driven DBFF, we systematically simplified the reconstruction requirements into two core components and three key geometric parameters (Figure 3). The two components comprised: (1) the superior vascularized fibula flap positioned to restore masticatory function and (2) the inferior 3D-printed porous titanium scaffold filled with autologous bone graft material to provide mandibular height and anatomical contour. The three critical parameters—L1, L2 and angle α-were derived from the contralateral healthy mandibular anatomy to guide fibular osteotomy planning.

**Figure 3:**
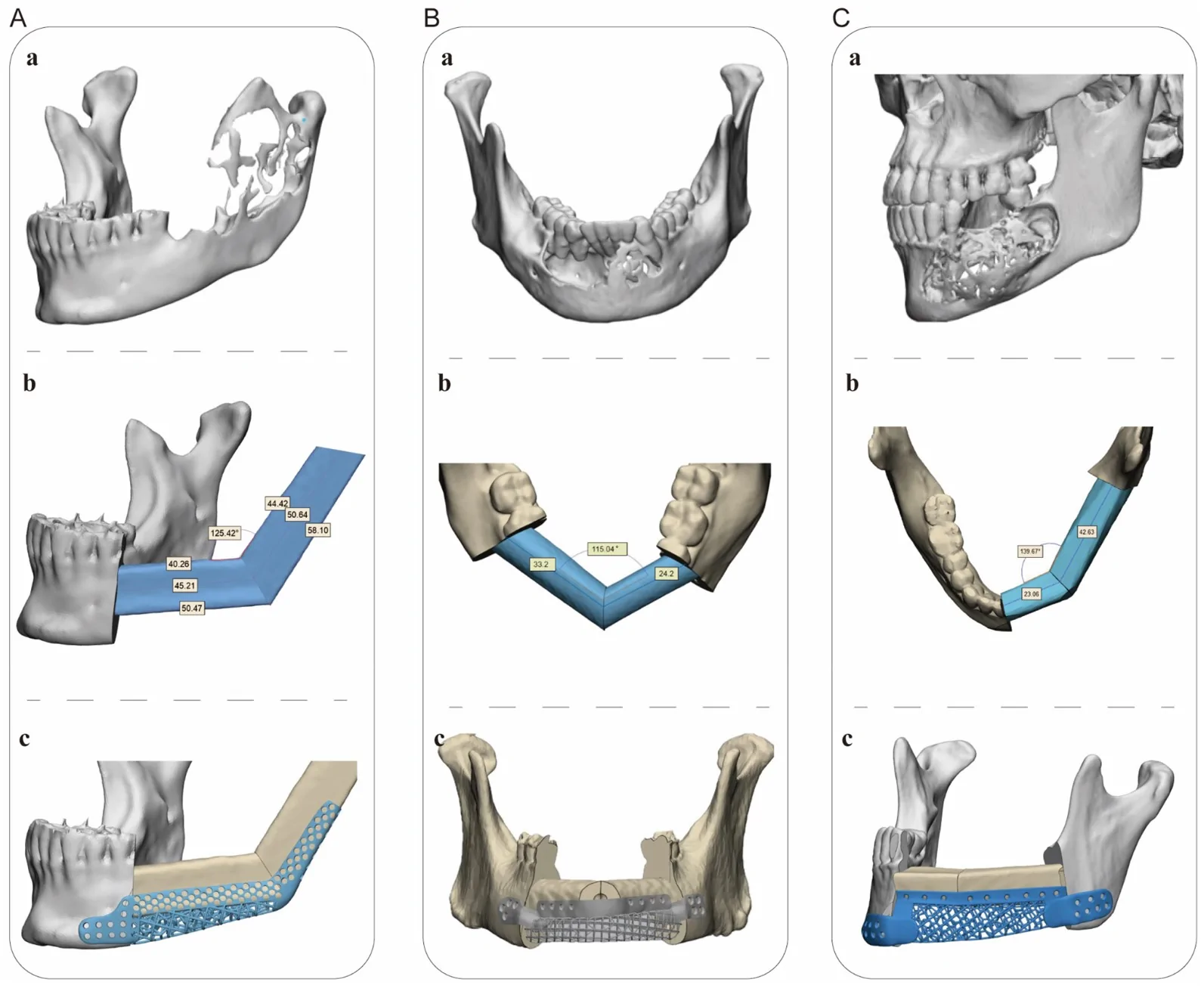
Structural design of scaffold-driven DBFF across three mandibular defect models. (**A**) The hemi-mandibular segment involving the condyle (HS: hemimandibular segment). (**B**) The central segment encompassing the canine teeth (CS: central segment). (**C**) The lateral segment excluding the condyle (LS: lateral segment). [(**a**) Reconstruction of CT data for mandibular lesions. (**b**) Mandibular defect simulated by fibular reconstruction. (**c**) Design of scaffold-driven double-barrel fibula flap.]

Based on the HCL classification system for mandibular defects, three representative defect models were established: hemimandibular segment involving the condyle (HS; Figure 3A), central segment encompassing the canine teeth (CS; Figure 3B) and lateral segment excluding the condyle (LS; Figure 3C). The scaffold design incorporated a biomimetic dual-layer architecture consisting of an external grid-like cortical shell (strut diameter: 0.5 mm) and an internal trabecular-like porous structure (pore diameter: 5.0 mm). This configuration achieved a porosity of approximately 90%, providing sufficient interspace for autologous bone graft integration while maintaining requisite mechanical strength. The design rationale was supported by previous experimental validation demonstrating favorable osseointegration with similar structural parameters. The large pore dimensions (5.0 mm) were specifically selected to accommodate bone graft materials and facilitate vascular ingrowth, contrasting with conventional smaller pore sizes (0.3–1.2 mm) that typically achieves only limited intra-scaffold osteogenesis.

Five strategic design considerations guided the scaffold-fibula integration: First, the fibula was positioned 2–3 mm inferior to the alveolar crest, accounting for soft tissue thickness (3–5 mm) to ensure adequate interridge distance and minimize exposure risk. Second, the scaffold was designed 1–2 mm medial to the native mandibular position to reduce scaffold exposure probability. Third, the fibular fixation plate was placed in the lower third of the fibula to avoid interference with subsequent dental implantation. Fourth, the mandibular fixation wing was engineered at 2.0 mm thickness with 2.0 mm screw holes to ensure sufficient mechanical fixation strength, while the fibular fixation wing measured 1 mm thickness with 1.5 mm screw holes, recognizing the lower initial stress on the fibula. Fifth, a 2.0 mm clearance was maintained between the fibula medial surface and the scaffold lingual side to facilitate intraoperative positional adjustments.

### Temporal evolution of stress distribution in scaffolds, fibula and mandibular bone

Finite element analysis revealed dynamic stress redistribution patterns across three critical time points: immediately post-implantation, 6 months post-implantation (pre-dental rehabilitation) and 9 months post-implantation (post-dental rehabilitation completed). The biomechanical behavior evolved significantly over time, with distinct temporal patterns observed for different anatomical structures (Figures 4 and 5).

**Figure 4:**
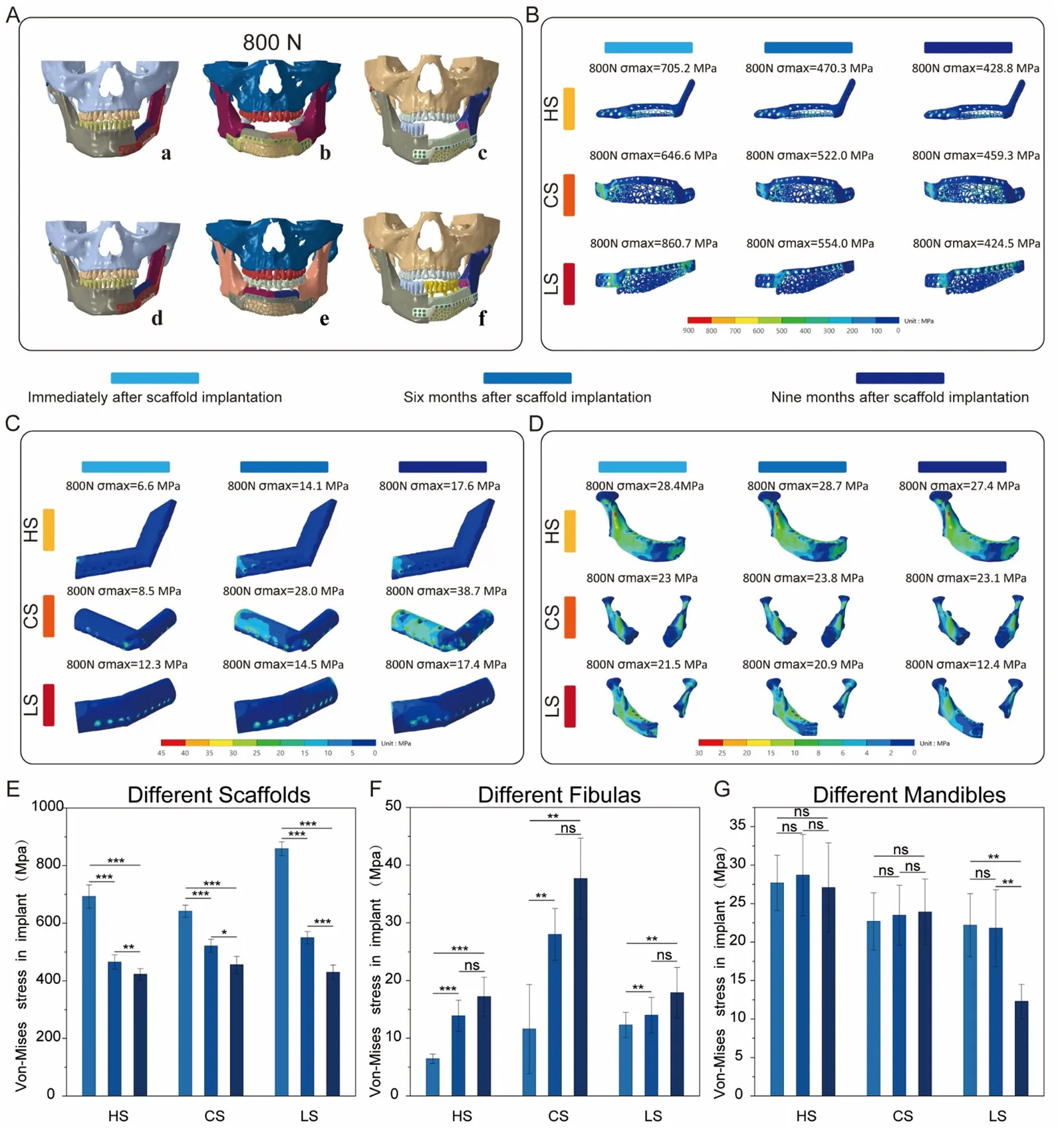
The process of stress distribution in the framework, fibula and mandible over time under a maximum biting force of 800 N. (**A**) The biomechanical models before and after denture restoration. (**a–c**) Biomechanical models before denture restoration and (**d–f**) biomechanical models after denture restoration. (**B**) von Mises equivalent stress of the 3D-printed titanium scaffold. (**C**) von Mises equivalent stress on the fibula. (**D**) von Mises equivalent stress on the mandible. (**E**) Comparison of von Mises equivalent stress over time in 3D-printed titanium scaffolds in different defect models under a maximum occlusal force of 800 N. (**F**) Comparison of von Mises equivalent stress over time in the fibula in different defect models under a maximum occlusal force of 800 N. (**G**) Comparison of von Mises equivalent stress over time in the mandible in different defect models under a maximum occlusal force of 800 N. Statistical significance was denoted as ns (not significant), *P < 0.05, **P < 0.01 and ***P < 0.001. Abbreviations: HS, hemimandibular segment; CS, central segment; LS, lateral segment.

**Figure 5:**
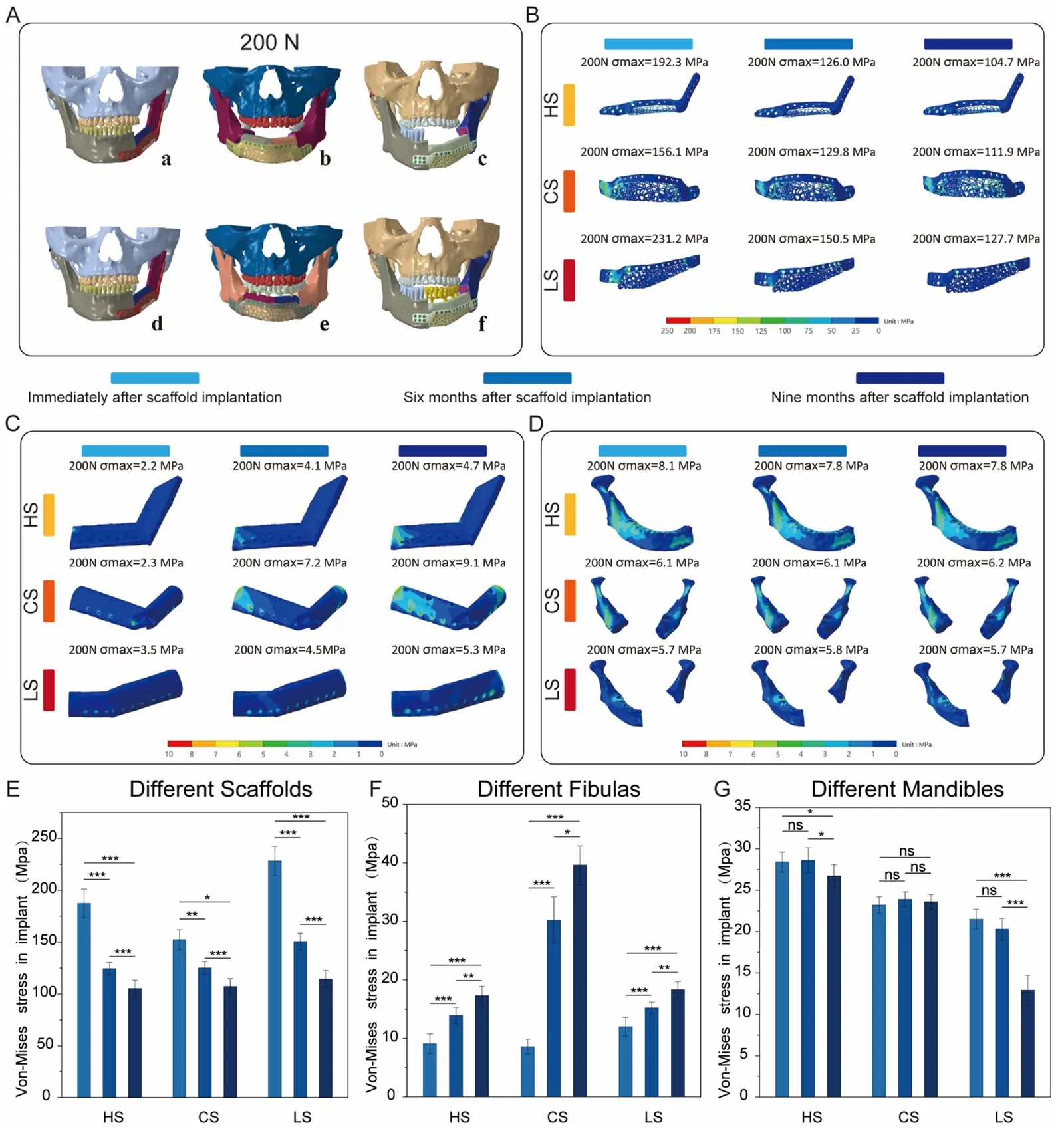
The process of stress distribution in the framework, fibula and mandible over time under a chewing force of 200 N. (**A**) The biomechanical models before and after denture restoration. (**a–c**) Biomechanical models before denture restoration and (**d–f**) biomechanical models after denture restoration. (**B**) von Mises equivalent stress of the 3D-printed titanium scaffold. (**C**) von Mises equivalent stress on the fibula. (**D**) von Mises equivalent stress on the mandible. (**E**) Comparison of von Mises equivalent stress over time in 3D-printed titanium scaffolds in different defect models under a chewing force of 200 N. (**F**) Comparison of von Mises equivalent stress over time in the fibula in different defect models under a chewing force of 200 N. (**G**) Comparison of von Mises equivalent stress over time in the mandible in different defect models under a chewing force of 200 N. Statistical significance was denoted as ns (not significant), *P < 0.05, **P < 0.01 and ***P < 0.001. Abbreviations: HS, hemimandibular segment; CS, central segment; LS, lateral segment.

The titanium scaffolds exhibited a consistent progressive stress reduction pattern across all three defect models throughout the 9-month observation period under both loading conditions **(**Figures 4B and E and 5B and E**)**. At immediate post-implantation time point, the scaffolds bore the highest stress levels under both maximum bite force (800 N) and physiological chewing force (200 N), with the LS model showing the greatest stress, followed by HS and CS, indicating that the rigid metallic scaffold served as the primary load-bearing structure. By 6 months post-implantation, a dramatic temporal decrease occurred across all models under both loading conditions, with approximately 21–56% reduction in maximum von Mises stress. This temporal stress reduction was statistically significant for all three models under both 800 N and 200 N conditions, indicating progressive load sharing between the scaffold and the osseointegrating fibula-mandible complex. Following dental rehabilitation at 9 months, scaffold stress further decreased across all models under both loading scenarios, with the temporal reduction from 6 to 9 months remaining statistically significant. Notably, inter-model differences became minimal at this final time point under both force magnitudes, with all three models achieving similar stress levels, suggesting that successful bone integration and occlusal restoration progressively normalized the biomechanical environment. The consistent temporal patterns observed under both 800 N and 200 N confirmed the load-dependent nature of stress distribution and the scaffold’s capacity to accommodate varying functional demands throughout the healing process.

The fibular grafts exhibited a temporally inverse stress pattern compared to the scaffolds, characterized by progressive stress increase over time under both loading conditions (Figures 4C and F and 5C and F). Immediately post-implantation, the fibula bore minimal stress across all three models under both 800 N and 200 N loading, while the rigid titanium scaffold dominated load transmission at this early stage. By 6 months post-implantation, the fibular stress substantially increased in all models under both loading conditions, with the temporal increase from immediate post-implantation to 6 months being statistically significant for all three models, representing an approximately 52–151% increase in fibular loading. At 9 months post-implantation with completed dental rehabilitation, the fibula assumed the majority of occlusal loading under both force conditions, with the temporal increase from 6 to 9 months remaining statistically significant, indicating continued biological integration and progressive load transfer from the scaffold to the vascularized bone graft. Notably, at this final time point under both 800 N and 200 N loading, the CS model demonstrated significantly higher fibular stress compared to both HS and LS models, attributable to concentrated masticatory forces during bilateral molar occlusion, while the HS and LS models benefited from residual natural dentition capable of bearing substantial occlusal forces. The inverse temporal relationship between scaffold and fibular stress across all three models under both loading magnitudes confirmed the successful biological integration process, wherein the initially rigid scaffold progressively transferred mechanical loads to the revascularized autologous bone over 9 months, ultimately achieving a biomechanically favorable load distribution that mimics native mandibular function.

In striking contrast to the dynamic temporal changes observed in the scaffold and fibula, the stress distribution in native mandibular bone remained remarkably stable across all three time points under both 800 N and 200 N loading conditions (Figures 4D and G and 5D and G**)**. Cortical bone stress values showed minimal temporal variation across the entire 9-month observation period under both loading magnitudes, with temporal comparisons revealing no statistically significant differences within each model. Similarly, cancellous bone stress remained within narrow ranges throughout the observation period under both force conditions, again with no significant temporal differences detected. This temporal stability of mandibular stress patterns under both loading conditions, despite substantial changes in scaffold and fibular loading, was attributable to the double-barrel structural design and the large contact interface between the scaffold and residual mandible, which effectively distributed loads and prevented stress concentration in the native bone throughout the entire healing and rehabilitation process. Both cortical and cancellous bone stress values remained within established physiological ranges at all time points under both loading conditions, indicating that the scaffold-driven DBFF successfully maintained normal bone homeostasis and avoided pathological stress shielding or overloading. The consistent temporal stability pattern across both maximum bite force and physiological chewing force validated the biomechanical compatibility and safety of this reconstruction approach throughout the critical healing period and subsequent functional loading phases.

### Biomechanical stability of bilateral temporomandibular joints

Assessment of bilateral articular disk stress provided critical insights into the functional integrity of the reconstructed mandible. The von Mises equivalent stress distribution in both right and left articular disks demonstrated remarkable temporal stability (Figure 6A–D), with minimal variation across the three observation time points under both chewing (200 N) and maximum bite force (800 N) conditions (Figure 6E–H).

**Figure 6:**
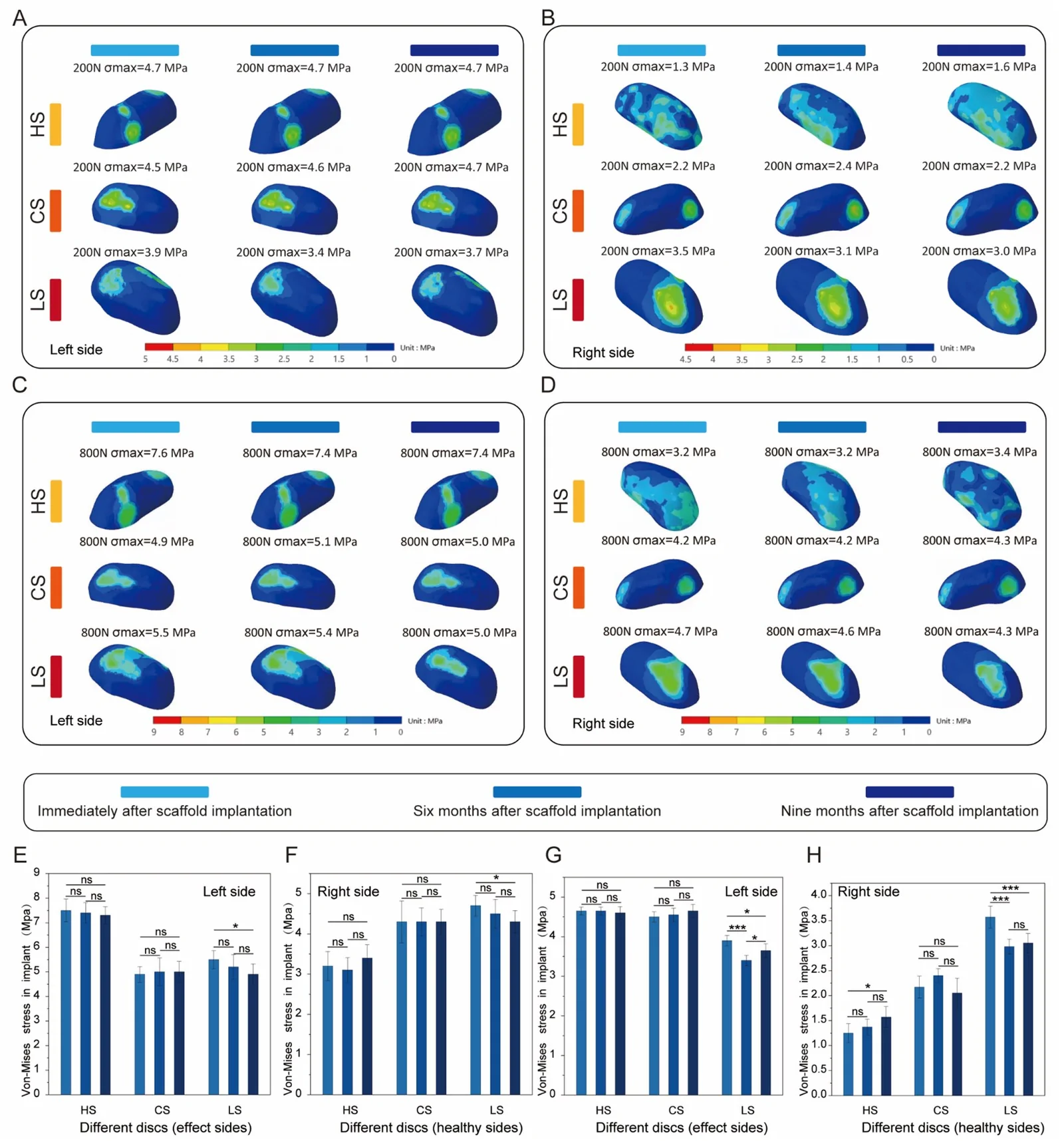
Bilateral temporomandibular joint stress distribution across three reconstruction models. (**A**) Right articular disk stress distributions across all three models under physiological chewing force (200 N). (**B**) Left articular disk stress distributions across all three models under physiological chewing force (200 N). (**C**) Right articular disk stress distributions across all three models under physiological chewing force (800 N). (**D**) Left articular disk stress distributions across all three models under physiological chewing force (800 N). (**E**) Quantitative comparison of left articular disc stress values across models and time points (200 N). (**F**) Quantitative comparison of right articular disk stress values across models and time points (200 N). (**G**) Quantitative comparison of left articular disc stress values across models and time points (800 N). (**H**) Quantitative comparison of right articular disc stress values across models and time points (800 N). Statistical significance was denoted as ns (not significant), *P < 0.05, **P < 0.01 and ***P < 0.001. Abbreviations: HS, hemimandibular segment; CS, central segment; LS, lateral segment.

The stress patterns remained consistent regardless of scaffold or fibular stress fluctuations, indicating that the scaffold-driven DBFF design successfully preserved normal temporomandibular joint biomechanics. This stability is crucial for preventing long-term joint complications such as disk displacement, degenerative changes or temporomandibular disorders.

The bilateral symmetry of stress distribution further confirmed that the reconstruction achieved balanced mandibular function, avoiding the compensatory loading patterns often observed with suboptimal reconstructions. This biomechanical equilibrium is essential for sustaining long-term masticatory efficiency and patient comfort.

### Postoperative accuracy and osseointegration assessment

Deviation analysis between preoperative digital planning and postoperative outcomes was conducted at 10 anatomical landmarks across bilateral sides (condyle, midpoint of posterior mandibular margin, mandibular angle, midpoint of inferior mandibular margin and coronoid process).

At 1 week postoperatively, substantial deviations were observed at all measurement sites, with mean displacement values ranging from 5.2 to 6.0 mm. The bilateral condyles showed deviations of 6.0 ± 1.5 mm (left) and 5.8 ± 1.1 mm (right), representing the maximum discrepancies. These early postoperative deviations were primarily attributed to acute soft tissue edema, which caused apparent but temporary positional shifts of the reconstructed mandible relative to the skull base reference points (Figure 7A and C).

**Figure 7:**
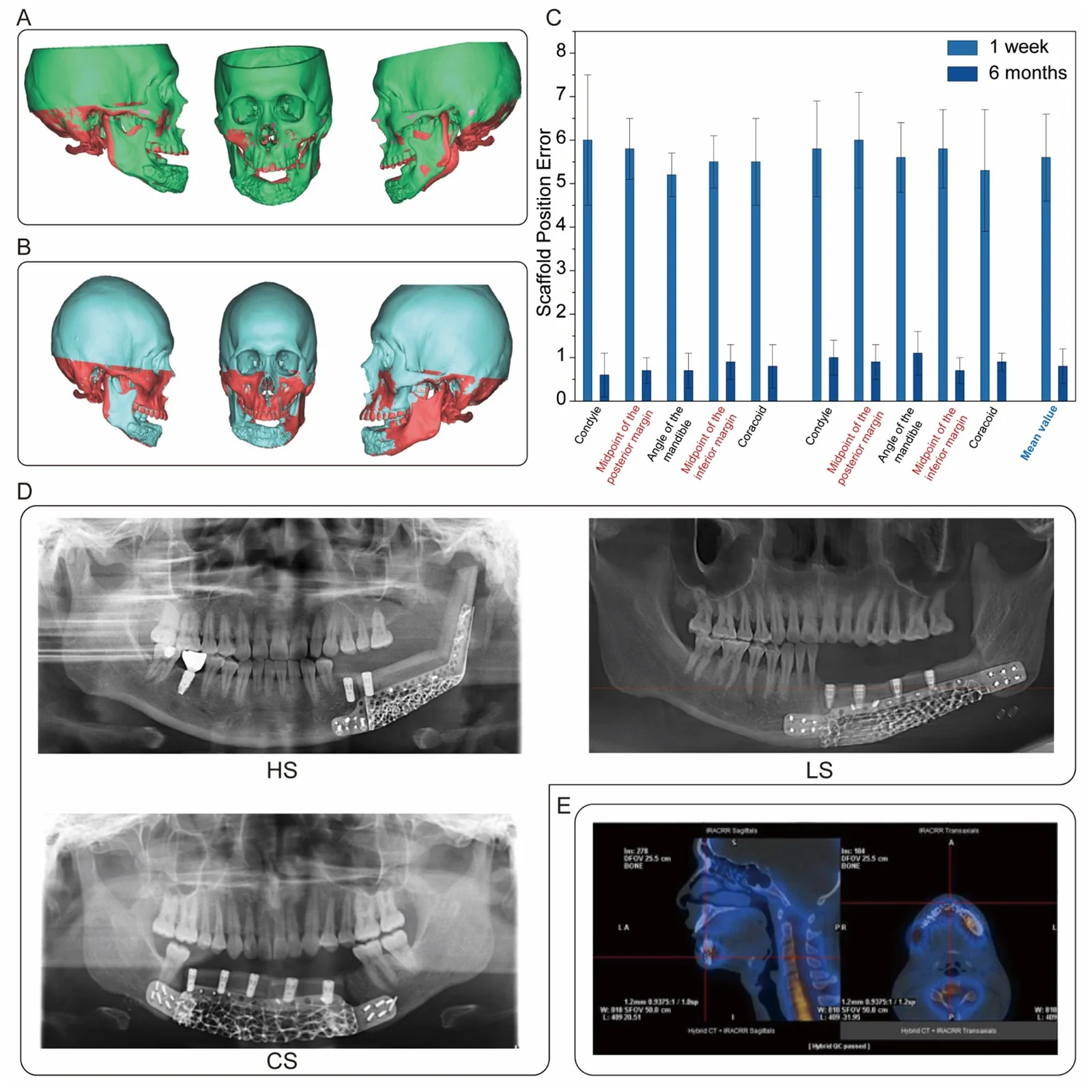
Postoperative deviation analysis and assessment of osseointegration. (**A**) Measurement of positional deviation between preoperative digital planning and postoperative geometry at 1 week. (**B**) Six-month positional evaluation showing substantial reduction in deviation after soft-tissue resolution and bone integration. (**C**) Error comparison between 1 week and 6 months post-surgery. (**D**) CT results demonstrating continuous trabecular bridging at the fibula–mandible junction. (**E**) ECT imaging confirming metabolic activity and intra-scaffold osteogenesis within the 5-mm porous titanium framework.

By 6 months postoperatively, following complete resolution of soft tissue edema and stable osseointegration, the deviations dramatically decreased to 0.8 ± 0.4 mm (range: 0.6–1.1 mm) across all measurement sites. This reduction was statistically significant and clinically meaningful, demonstrating excellent concordance between planned and achieved reconstruction geometry. The condylar positions showed particularly favorable accuracy, with deviations of only 0.6 ± 0.5 mm (left) and 1.0 ± 0.4 mm (right), confirming precise restoration of mandibular ramus height and temporomandibular joint relationships (Figure 7B and C).

Computed tomography (CT) and emission computed tomography (ECT) examinations at 6 months postoperatively provided compelling evidence of successful osseointegration and intra-scaffold bone regeneration (Figure 7D and E). Radiographic analysis revealed smooth osseointegration at the fibula-mandible junction, with continuous trabecular bridging across the bone-implant interface. More notably, ECT examination demonstrated active metabolic activity within the porous titanium scaffold, indicating ongoing osteogenesis throughout the scaffold volume rather than merely at the peripheral bone-implant interface. This intra-scaffold osteogenesis represented a critical advancement over previous titanium implants, which typically achieved only superficial osseointegration with minimal internal bone ingrowth. The large pore dimensions combined with autologous bone graft filling created a favorable biomechanical and biological environment that promoted complete volumetric bone regeneration.

The successful osseointegration significantly reduced the risk of long-term mechanical complications associated with permanent metallic implants. By transitioning from a metal–skin interface to an autologous bone–skin interface through progressive bone ingrowth, the reconstruction achieved enhanced soft tissue stability and reduced scaffold exposure risk.

### Clinical outcomes and patient-reported satisfaction

Sixteen patients underwent scaffold-driven DBFF reconstruction for mandibular defects, representing three defect models: HS (*n* = 7), CS (*n* = 5) and LS (*n* = 4; Table 2). The mandibular defect lengths ranged from 21.3 mm to 49.2 mm, requiring corresponding fibular segment lengths with osteotomy angles ranging from 98.41° to 145.32°.

**Table 2:** Postoperative follow-up and patient satisfaction.

No.	Age (years)	Sex	Model	Fibula length (mm)^a^	Fibula angle (°)	Operative time (min)	Follow-up period (months)	Patient satisfaction (1–5)
1	30	F	HS	45.88/52.00	121.66	370	21	4.7
2	41	M	LS	23.06/42.63	139.67	390	17	4.8
3	24	M	HS	46.23/51.88	127.81	450	18	4.5
4	31	M	HS	47.82/49.76	120.42	380	20	4.2
5	21	M	CS	33.20/24.20	115.04	410	12	4.3
6	44	M	CS	28.77/34.53	99.26	500	9	4.5
7	62	F	CS	33.66/33.40	98.41	420	9	4.7
8	28	M	LS	25.26/41.35	145.32	380	13	4.3
9	19	F	HS	43.81/48.54	123.45	360	12	4.8
10	35	F	CS	34.25/33.16	110.24	380	14	4.6
11	27	M	HS	46.12/50.39	119.36	420	28	4.9
12	46	F	LS	22.35/39.78	139.78	450	22	4.6
13	52	F	HS	44.78/52.13	125.42	420	13	4.7
14	39	M	LS	27.68/38.21	142.37	430	11	4.5
15	43	M	HS	49.21/47.19	127.14	390	16	4.6
16	47	M	LS	21.34/42.56	144.25	400	18	4.5
Average	38	–	–	–	–	410.8	15.2	4.6

a For DBFFs, values separated by “/” represent measurements of the superior and inferior fibular segments, respectively.

The mean operative time was 410.8 min (range: 360–500 min), which compared favorably with traditional DBFF procedures requiring complex double-barrel folding and multiple vascular anastomoses. The mean follow-up period was 15.2 months (range: 9–28 months), providing sufficient duration to assess both short-term surgical success and intermediate-term functional outcomes.

Dental rehabilitation was completed at 9 months postoperatively for all patients, with postoperative intraoral photography and radiographic imaging demonstrating excellent restoration of facial contour and occlusal relationships (Figure 8A). The scaffold-driven DBFF successfully addressed the vertical height discrepancy between native mandible and fibula, providing adequate bone volume for dental implant placement without requiring additional vertical bone augmentation procedures.

**Figure 8:**
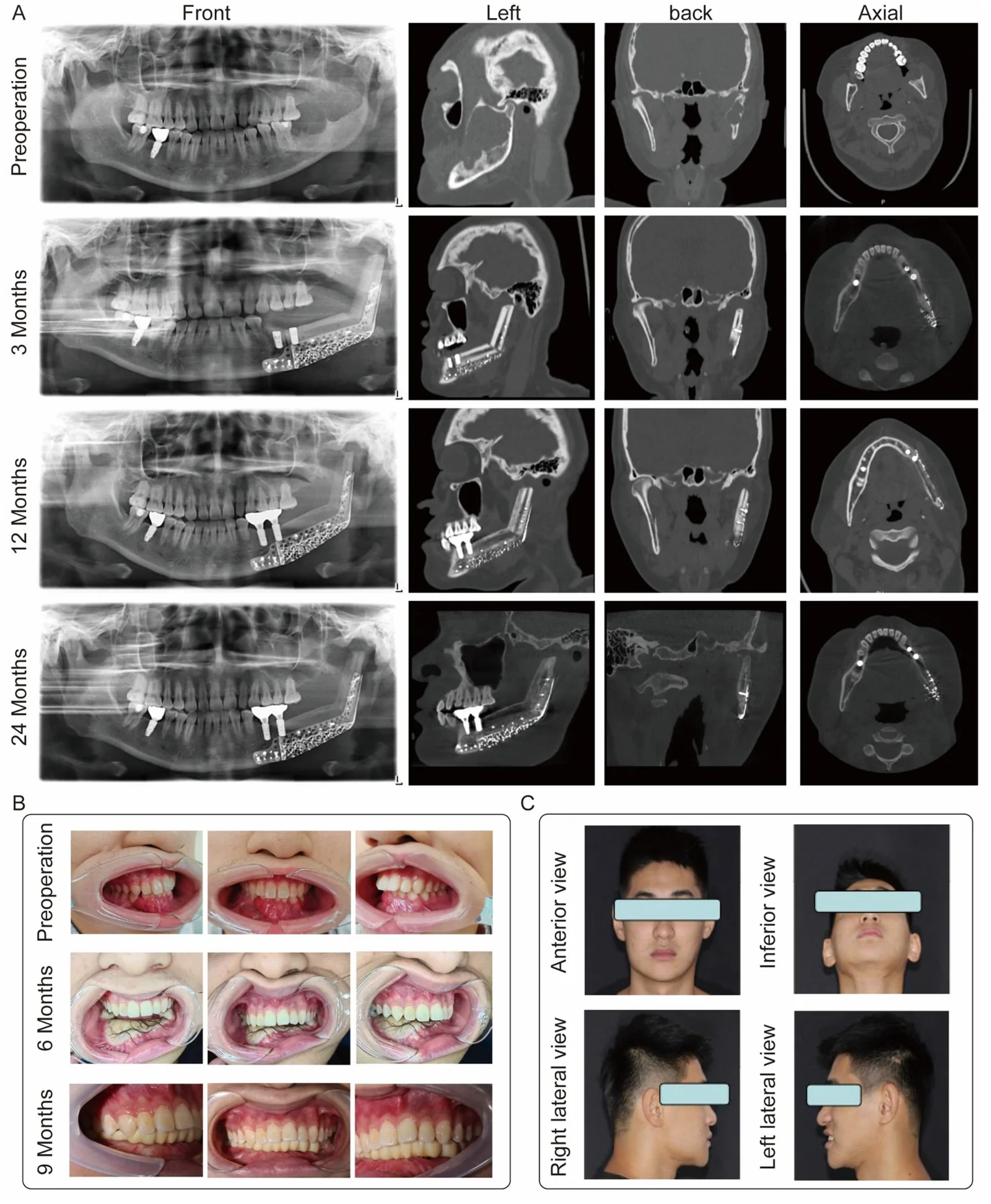
Clinical outcomes and postoperative functional rehabilitation. (**A**) Images taken 24 months after the patient’s DBFF procedure show that tissue and function have recovered and are maintaining a good condition. (**B**) Oral examination images of the patient within 9 months after DBFF surgery. (**C**) Postoperative facial appearance demonstrating restored contour and symmetry.

Patient satisfaction, assessed using a 5-point Likert scale across six functional domains (facial symmetry, facial height, articulation, chewing, swallowing and mandibular movement), achieved a mean score of 4.6 (range: 4.2–4.9). This high satisfaction level reflected successful restoration of both esthetic appearance and oral function. Specifically, patients reported near-complete normalization of mastication, speech articulation and swallowing function, with only minor perceived differences from pre-pathological baseline function (Figure 8B and C).

No major mechanical complications (scaffold fracture, severe loosening or catastrophic failure) were observed during the follow-up period. Minor complications were limited and manageable, demonstrating the clinical safety and reliability of this reconstruction approach. The absence of mechanical failures validated the finite element-optimized scaffold design, which successfully balanced mechanical strength requirements with biological bone regeneration objectives.

Importantly, no cases of severe scaffold exposure requiring surgical revision were documented. The strategy of filling the porous scaffold with autologous bone graft material, which progressively transformed the metal-soft tissue interface into a bone-soft tissue interface through internal osseointegration, proved effective in preventing exposure complications commonly associated with permanent metallic implants.

## Discussion

In the present study, we designed a scaffold-driven DBFF using 3D-printed titanium scaffolds and autologous fibula flaps based on preoperative biomechanical and digital data. This innovation offered an excellent functional recovery from mandible bone defects, a successful quick dental implantation and a high accuracy of implant placement. With the guidance of preoperative biomechanical evaluation and digital design, our surgical scheme provided an adequate height for dental restoration while preserving the esthetic contours of the mandible. Meanwhile, sufficient biological stimulation from bone graft materials could initiate bone healing immediately.

Autologous bone grafting is the gold standard for repairing mandibular defects and reconstructing the mandible, boasting an evident osteogenic effect and long-term functional stability [12, 22]. A DBFF has been proven safe and effective to repair mandibular defects, increase the height of mandible and restore the facial shape. Although a 25-cm-long fibula can be utilized, DBFF is not recommended to repair large bone defects (>10 cm) considering the removal of bone mass in the folded fibula and preservation of bone mass for vascularized pedicle [23, 24]. In addition, a DBFF is not always reliable for creating an ideal height of bone graft. It is found that DBFF achieves a larger bone height that hinders the following dental implantation [25, 26]. Here, free vascularized fibular flaps were used to ensure the survival of osteoblasts and viability of tissues after transplantation. Previous studies have demonstrated that these flaps are particularly suitable for mandibular reconstruction after trauma or tumor resection [27], yielding a high survival rate of the bone graft [28, 29]. The outcomes in the present study consistently verified their reparative effects.

Our previous findings have shown the application of 3D-printed titanium grid scaffold in treating three cases of mandibular segmental defects, although the efficacy should be further validated due to the short-term follow-up [18]. One case was postoperatively managed by two modified surgical procedures for the same myxofibroma on the mandible. Mandibular bone defects caused by tumors, trauma or osteoradionecrosis present varying locations and extents [30]. A customized mandibular reconstruction is expected to maximize the recovery of facial shape, swallowing movements and language functions [31]. A panel of modern medical techniques, like 3D printing, application of biomaterials and stem cell tissue engineering has been frequently applied to repair mandibular bone defects [32, 33].

Considering the various forces borne by the 3D-printed scaffolds in repairing bone defects at different parts, we classified mandibular defects into three categories based on the HCL classification for hemimandibular (H), central (C) and lateral (L) defects [34]. Our biomechanical data showed a gradually decreased von Mises equivalent stress of the scaffolds over time. Among them, LS produced the greatest von Mises equivalent stress at the early stage, reaching 860.7 MPa at the maximum bite force of 800 N. All three categories of mandibular defects presented a nearly 50% of von Mises equivalent stress decrease (400–500 MPa) after the scaffold repair.

In the early stage, the 3D-printed titanium scaffolds were dominantly responsible for bearing the von Mises equivalent stress of mandibular defects of the CS and LS types. The fibula barely bore the bite force. On the contrary, the HS undertook a significantly larger stress than the CS and LS in the early stage, due to the need to maintain the shape of the ramus of the mandible and bear the stress on the joint area. After bone healing at 6 months and dental implantation, CS bore a high stress than the HS and LS, because of the greater bite force, while the remaining natural teeth in the latter two models were capable of bearing more occlusal force. In addition, a well-designed repair regimen was superior in achieving a stable stress load on the articular disk and mandible, with little variation over time.

In the present study, we measured the distribution of von Mises equivalent stress on the titanium scaffold, fibula, mandible and bilateral articular disks. Immediately after dental implantation, the stress on the mandible caused by the bite force was almost all undertaken by the titanium scaffold. After 6 months of implantation, the occlusion was further reduced by the titanium scaffold, and the fibula borne the majority of the bite force. The mandible was subjected to less and stable mechanical stress, probably due to the double-barrel structure of the titanium scaffold and a large interface between the mandible and scaffold [35, 36]. It should be noted that the fixation screws were not explicitly included in the current finite element model. Therefore, localized stress behavior at the screw–bone interface could not be directly evaluated in this study. Nevertheless, the overall stress distribution of the reconstructed mandible–fibula–scaffold system demonstrated stable load transfer without abnormal global stress concentration, indicating satisfactory mechanical performance of the construct under physiological loading conditions.

In addition to the biomechanics of reconstructed mandible, postoperative deviation between the planned and actual positions of the implant is also an important indicator to reflect the success or failure of mandibular reconstruction. The position of reconstructed mandible visualized by CT scans directly influences the functional recovery [37]. In comparison to the preoperative digital design, we virtually positioned 10 landmarks of the mandible on bilateral sides at 1 week and 6 months postoperatively. A large deviation at 1 week postoperatively may be attributed to local soft tissue edema. With the gradual retreat of edema, the displacement was largely reduced. On the postoperative imaging scans, landmarks on the skull were referred to for evaluating the deviation between planned and actual positions. A large deviation could also be caused by a relative displacement of the mandible relative to the skull.

Existing porous titanium implants, usually presenting a pore size ranging from 0.3 mm to 1.2 mm, have been experimentally validated for their favorable outcome in promoting local osseointegration [38]. Although porous materials can achieve osseointegration and partial osseointegration, only a small number of osteogeneses can satisfy the complete osteogenesis in the scaffold [39, 40]. Here, we allowed a larger pore size (5.0 mm) in the 3D-printed titanium scaffolds to functionally repair the mandibular bone defect. Through optimizing the mechanical properties, our 3D-printed titanium scaffolds with a larger pore size effectively reduced the amount of titanium alloy and offered sufficient space for bone grafting. The excess fibula during surgery was implanted into the mesh of the scaffold to achieve the goal of osteogenic repair. CT and ECT examinations at 6 months postoperatively proved the smooth osseointegration of the fibula and mandible, thus reducing the risk of complications due to a long-term mechanical implantation. In addition, filling of autologous excess fibula combined with local osteogenesis altered the biomechanical composition of the interface between the scaffold and skin/soft tissue. The change from the metal-skin contact to an autologous bone-skin interface effectively prevents the scaffold from being exposed after breaking through the skin. Overall, a larger pore size of the titanium scaffolds was conductive to dispersing the occlusal force and reduce the stress on the restoration and the occurrence of complications.

In the future, *in vitro* and *in vivo* experimental studies are warranted to further elucidate the osteogenic mechanisms at the interface between 3D-printed titanium scaffolds and vascularized fibular flaps in mandibular defect reconstruction. In particular, histological and molecular analyses during the healing process may provide deeper insights into vascularization patterns, osteogenic differentiation and bone–implant integration dynamics. In addition, multicenter, large-scale clinical trials are still required to further validate the long-term efficacy, safety and generalizability of this scaffold-driven reconstruction strategy across diverse patient populations.

Importantly, it should be noted that the present study focuses primarily on clinical translation and biomechanical validation; therefore, detailed mechanistic investigations at the cellular and molecular levels were beyond its scope. These aspects will be addressed in future work to further strengthen the biological foundation of this reconstruction strategy.

In conclusion, the scaffold-driven DBFF strategy using 3D-printed porous titanium scaffolds combined with vascularized fibular flaps provides an effective approach for the reconstruction of segmental mandibular defects. This method enables reliable restoration of mandibular continuity with reduced autologous bone harvesting, favorable safety profiles, satisfactory esthetic outcomes and biomechanically stable load transfer that closely mimics physiological mandibular mechanical behavior. Overall, this digitally designed and biomechanically guided reconstruction strategy demonstrates promising translational potential for clinical application in complex mandibular defects.

## Data Availability

Data supporting the findings of this study are available within the article and its supplementary information files and from the corresponding author on reasonable request.
